# Development of collaboration guidelines for nursing education and related healthcare services

**DOI:** 10.4102/hsag.v29i0.2496

**Published:** 2024-02-16

**Authors:** Estelle Bester, Neltjie C. van Wyk, Carin Maree

**Affiliations:** 1 Department of Nursing Science, Faculty of Health Sciences, University of Pretoria, Pretoria, South Africa

**Keywords:** nursing education institution, associated healthcare services, clinical facilitators, theoretical lecturers, theory and practice integration

## Abstract

**Background:**

A theory-practice gap in nursing education often occurs, and the staff from the nursing education institution and the associated healthcare services should find ways to improve their collaboration to reduce the gap during the training of nursing students.

**Aim:**

This study aimed to develop context-specific collaboration guidelines for a nursing education institution and associated healthcare services.

**Setting:**

Private hospital in the Gauteng province of South Africa.

**Methods:**

Guidelines were developed from the findings of an integrative literature review. Thereafter, it was contextualised in a qualitative study with focus group discussions (FGDs) involving 9 theoretical lecturers and 10 clinical facilitators.

**Results:**

In the partnership between the nursing education institution and the associated healthcare services, bilateral communication, cooperation between the theoretical lecturers and the clinical facilitators in delivering evidence-based patient care, intensified innovation in teaching and learning practices and an environment conducive to theory-practice integration should be emphasised.

**Conclusion:**

A set of context-specific guidelines was developed to enable the theoretical lecturers and the clinical facilitators to collaborate in supporting nursing students to apply their theoretical knowledge in the development of clinical competencies.

**Contribution:**

The guidelines can be adjusted to suit the context of other nursing education institutions and their associated healthcare services to improve collaboration between theoretical lecturers and clinical facilitators to the benefit students’ skills development in theory-practice integration.

## Introduction

Nursing students often face confusion when there is a mismatch between theoretical knowledge and its application in real clinical settings (Factor, Matienzo & De Guzman 2017:84; Kerthu & Nuuyoma 2019:24). The practical experience in healthcare services often differs from the idealised scenarios presented in theory classes. While simulation laboratories are well-equipped with the necessary resources, the associated healthcare services often lack such resources (Mina, Reza & Dimitrios 2019:403). As a result, students frequently struggle to implement what they have learned in theory into rendering care to patients (Greenway, Butt & Walthall 2019:5). Poor collaboration between theoretical lecturers and clinical facilitators contributes to the challenges that students experience to integrate theory in practice (Günay & Kılınç 2018:84). It is crucial for theoretical lecturers and clinical facilitators to collaborate effectively to support students in bridging theory-practice gap.

This study aimed to develop context-specific collaboration guidelines for the theoretical lecturers of a designated nursing education institution and the clinical facilitators of its associated healthcare services. To gain data for the development of the guidelines, a comprehensive literature review on collaboration between theoretical lecturers and clinical facilitators to improve theory-practice integration was conducted. The guidelines were then contextualised with the input of theoretical lecturer and clinical facilitator participants.

## Research methods and design

**FIGURE 1:**
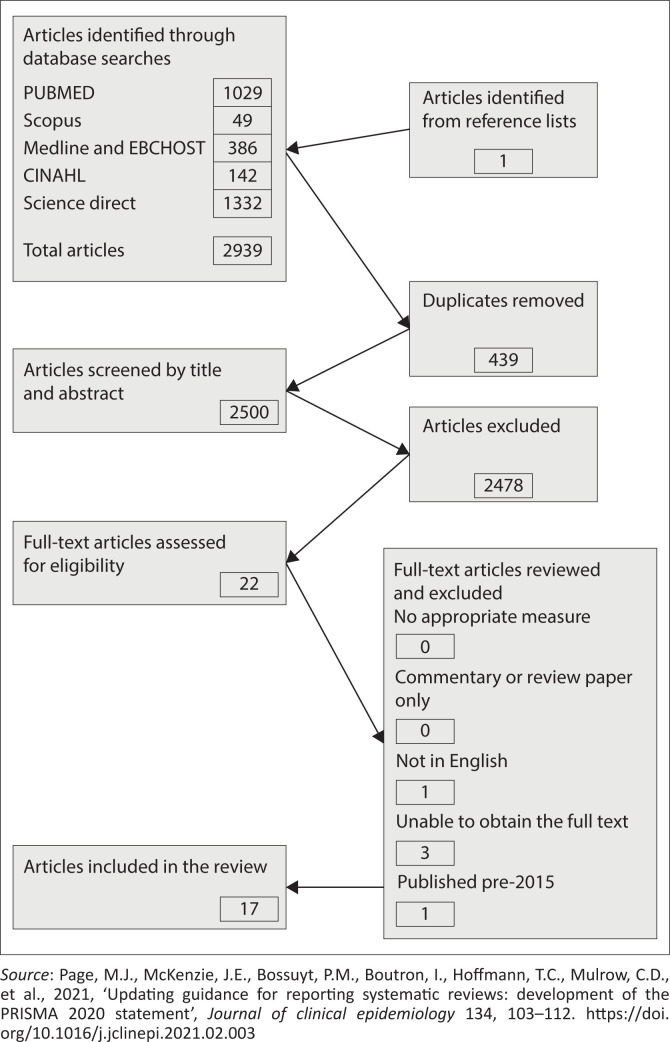
Articles selected for review.

The research was done in two phases. In the first phase, the researcher conducted a literature review using five databases: PubMed, Science Direct, Medline and EBCHOST, CINAHL and Scopus, to collect evidence on good collaboration practices between nursing education institutions and healthcare services, particularly between theoretical lecturers and clinical facilitators, to enhance theory-practice integration during clinical training. The search led to the selection of 2939 articles, which were then screened. After removing duplicates and excluding articles that were not related to collaboration in nursing education, 47 full-text articles were reviewed. Finally, 17 articles met the inclusion criteria of being published in English, having full-text availability, and being peer-reviewed publications. These articles were published between January 2016 and January 2021 (refer to Figure 1).

The first author conducted a thematic analysis of the findings. It involved a familiarisation with the content and searching for themes. She also drafted collaboration guidelines from each article (refer to Table 1). The summarised articles and themes formed the basis for the draft guidelines.

**TABLE 1:** Summary of the articles.

References	Study aim	Design, methods, setting, participants	Key findings of the study	Guidelines drafted from the publication
Akram, A.S., Akram, S. & Mohamad, A., 2019, ‘The role of clinical instructors in bridging the gap between theory and practice in nursing education’, *International Journal of Caring Sciences* 11(2), 876–882.	The study aimed to assess the presented role of clinical nurse instructors in bridging the gap between theory and practice from the perspective of student nurses.	Descriptive quantitative cross-sectional design; questionnaire using a Likert scale; governmental hospitals in Gaza; 135 nursing student participants in 3rd and 4th years of training	Clinical instructors should be present in the hospitals to help students to bridge the theory-practice gap; have good communication skills; use time effectively when planning training activities; familiarise themselves with the theoretical content that is taught in the nursing institution.	Consolidate clinical teaching and learning strategies and provide effective supervision and feedback to foster students’ confidence and professional development. Create a structured academic and clinical operational plan to support theory-practice integration with knowledge and skills development. Include clinical facilitators during theoretical classes to help link clinical scenarios with the teaching of theory during lectures. Create a communication system to ensure adequate and timely communication between the clinical team and academic lecturers for weekly theoretical outcomes to connect or link to the theory that is done at the nursing campus.
Bay, E.H. & Tschannen, D.J., 2017, ‘An academic-service partnership: A system-wide approach and case report’, *Journal of Nursing Education* 56(6), 373–377.	This study aimed to report an overview of the first year of full implementation, and survey results from nurse leaders and faculty on the academic service partnership.	Case report; nursing leaders, lecturers and clinical mentors	Academic and service leadership should support the implementation of clinical teams. Clinical teams consisting of clinical mentors, lecturers and students, focus on patient care and educational outcomes. The clinical mentors support students while caring for patients; self-directed learning encouraged. Lecturers need to determine if learning goals were achieved and can expand learning opportunities.	Developing clinical mentors is crucial to support students during clinical practical sessions. Providing patient care and achieving students’ outcomes should be integrated and the focus of a clinical team. Provide information to clinical teams on students’ academic outcomes and the process of achieving the outcomes. Implement a communication system to ensure adequate and timely communication between: Clinical facilitators and lecturers for weekly discussions of clinical outcomes and to link theory and clinical training; and clinical teams and students for feedback regarding progress.
Berndtsson, I., Dahlborg, E. & Pennbrant, S., 2020, ‘Work-integrated learning as a pedagogical tool to integrate theory and practice in nursing education - An integrative literature review’, *Nurse Education in Practice* 42, 1–8.	This integrative literature review aimed to identify models for the integration of theory and practice during clinical placements in nursing education by using work-integrated learning.	Integrative literature review; 16 articles	Students need positive role models, appreciate feelings of belongingness, want to experience peer support, need opportunities to develop critical thinking abilities. Clinical supervision of students may enable their critical thinking skills; identify students’ learning needs; help them to solve problems and to integrate theory into practice through improved clinical reasoning skills. Link research to practice development and practice development to research.	Develop a structured orientation programme for lecturers and clinical facilitators that include: Pedagogical teaching practices for example, reflection and clinical reasoning cycle and problem-solving skills; the use of digital resources during clinical learning to integrate theory in practice; giving feedback to students to enhance learning. Encourage clinical facilitators to use reflection, problem-solving and clinical reasoning cycles in clinical practices. Provide opportunities for students to present case studies to clinical facilitators and lecturers. Involve clinical facilitators during theoretical classes to help link clinical scenarios of the hospital with theory in class. Involve lecturers during clinical facilitation to help link theory to clinical cases in the hospital.
Bvumbwe, T., 2016, ‘Enhancing nursing Education via an academic-clinical partnership: Integrative review’, *International Journal of Nursing Sciences* 3(3), 314–322.	Describe the role of academic-clinical partnerships in strengthening nursing education.	Integrative review; 33 articles	Academic and practice colleagues need to collaborate for a common goal through mutual understanding of responsibilities, shared values and goals to enhance a learning culture for theory-practice integration in evidence-based practice. It includes staff development.	Create an academic practice partnership with a common goal of: Development of mentor programmes for capacity building of clinical facilitators and lecturers; creating of learning culture and environment; sharing of responsibilities between two systems. Facilitate collaboration between clinical facilitators and lecturers with planed regular meetings to discuss problems and strategies to improve the partnership.
Clevenger, C.K. & Cellar, J., 2018, ‘Creating new models of care through an academic-clinical partnership’, *Georgia New Administration Quarterly* 42(4), 30–31.	To describe an exemplar of academic clinical partnership in which a new model of care is co-designed and co-produced with positive results for patients, care partners and the health system.	Essay; Nell Hodgson Woodruff school of Nursing; Emory University School of Medicine department; and primary advanced healthcare clinic	The Integrated Memory Care Model was developed around clinical practices for: Comprehensive primary care, individualised care plans; aggressive symptom assessment; thoughtful utilisation of healthcare and community-based services delivered by a clinical team of high-functioning health professionals.	Utilised new and different clinical care models and clinical practice guidelines to improve student knowledge application, professional development and employability.
Dev, M.D.B., Rusli, K.D.B., McKenna, L., Lau, S.T. & Liaw, S.Y., 2020, ‘Academic-practice collaboration in clinical education: A qualitative study of academic educator and clinical preceptor views’, *Nursing and Health Sciences* 22(4), 1131–1138.	To study the perceptions of academic educators and preceptors on their collaboration in the transition to a practice programme.	Qualitative explorative study; interviews with 12 preceptors and 13 academic educators of the National University of Singapore and 3 academic hospitals	Efficient communication between preceptors, clinical instructors and academic educators is important for collaboration. All stakeholders need to be involved in the development of learning objectives and structured learning and practice guidelines to support students. Preceptors and academic educators need to be familiar with each other’s responsibilities.	Create a direct communication structure between academic lecturers and clinical facilitators to communicate student objectives, and feedback on identified learning needs and concerns. Implement an orientation programme for newly appointed clinical facilitators regarding the learning programme or curriculum, practice guidelines of the hospital, and required level of competence during clinical assessment. Joined clinical assessments between clinical facilitators and lecturers, to ensure the quality of assessments and consistence in teaching and learning. Employ alumni staff from nursing intuition to support students in a clinical setting as preceptors.
Drayton-Brooks, S.M., Gray, P.A., Turner, N.P. & Newland, J.A., 2017, ‘Building clinical education training capacity in nurse practitioner programmes’, *Journal of Professional Nursing* 33(6), 422–428.	To explore new clinical education training models for nurse practitioners.	Essay; PENN Medicine GNE Partnership; Philadelphia; United States	Lecturers need to interact with preceptors and evaluate students’ clinical competency. Inter-professional collaboration of lecturers and the involvement of students from different year groups may contribute to quality clinical training. Shared goals regarding teamwork, communication and values may contribute to efficient inter-professional collaboration. Maximising academic faculty practice partnerships to provide students with well-coordinated clinical experiences in evidence-based practice. Use electronic portal resources for communication.	Promote student placement in different care settings for comprehensive understanding and application of nursing knowledge and skills (e.g., Geriatrics, long-term care facilities, weekend support in community clinics, convenience care clinics). Intensify clinical training with a focus on speciality training and assessment in specialise areas (e.g., medication administration and handling, infection control, mother and baby wellness, theatre safety). Plan and collaborate with other health professionals to integrate curriculums to expand students’ learning experiences, promote teamwork and communication, and understand each role and responsibility. Build clinical training capacity within services to improve stakeholder relationships through preceptor development, simplify the student evaluation process and assurance of student readiness for practice.
Hussein, M.T.E.L. & Osuji, J., 2017, ‘Bridging the theory-practice dichotomy in nursing: The role of nurse educators’, *Journal of Nursing Education and Practice* 7(3), 20–25.	Discussion paper to shed light on the roots of theory practice dichotomy and suggest some strategies that may bridge this gap.	Discussion paper	Optimal communication between professional nurses in practice and nurse educators required for sharing of information regarding the clinical training of students. Students’ reflective skills, critical thinking and reflective practice capacity need to be developed to help close the theory knowledge and practical skills gap.	Incorporate with-in the partnership goals: Capacity building nursing programmes to enrich professional nurses’ knowledge in hospital settings, Open communication channels for sharing of nursing knowledge between nursing education and clinical settings; Teaching of reflective thinking skills to nurses.
Gierach, M., Knuppe, M., Winterboer, V. & Randall, R., 2019, ‘Creating a culture of caring: A collaborative academic-practice approach to clinical education’, *Nursing Forum* 54(3), 386–391.	Describe the implementation of the clinical education model of Culture of Caring.	Implementation of a clinical model in a team-based approach and collaboration between academic and clinical staff; 3 large academic institutions; academic practise leaders, clinical instructors, staff nurses and students	Clinical instructors help students to integrate theory into practice by facilitating clinical learning. Academic-clinical partnerships need to have a shared curriculum to guide the clinical experience. They need to agree on clinical assignments. Teamwork improves the relationship between stakeholders where knowledge can be shared in safe environments. Lecturers should visit the clinical facilitators and offer support with students’ endeavours with theory-practice integration. A learning conducive clinical environment may enable students to develop clinical skills.	Creation of structured clinical education models between clinical facilitators, lecturers, preceptors and students, to create a positive learning environment, teamwork and communication and support theory-practice integration. Provide orientation to preceptors and students regarding roles and responsibilities, expectations and requirements with in units. Support preceptors in the unit through relationship building, mentoring in a clinical setting and regular meetings. A shared curriculum within the academic-clinical partnership needs to guide and support the integration of academic curricula.
Gursoy, E., 2020, ‘The partnership between academic nursing and clinical practice: A qualitative study’, *Journal of the Pakistan Medical Association* 70(4), 597–601.	To assess the impact of a partnership between a nursing school and a largescale urban hospital on health education, practice and research.	Qualitative study; In-depth interviews using opened questions; 16 faculty members 8 nurse clinicians; Qualitative content analysis; Urban hospital and nursing school in Pennsylvania, Philadelphia	Leadership is a key factor for a successful partnership. Mutual cooperation, communication and clearly defined goals between academic staff and practice staff may enhance understanding of each system and improve the quality of teaching in a positive learning environment. Clinical educators play a dual role in the partnership by being innovative in sharing of knowledge to both sides to enhance understanding of each other’s responsibilities. They need to be research orientated to support staff to solve nursing problems and have positive patient outcomes.	Create a mutual partnership with a broad vision, clear goals and a good communication system to support staff, and benefit quality standards of both systems. Partitive collaborative research practice to enhance academic staff, skills and knowledge transferability in a clinical and academic setting.
Huston, C.L., Phillips, B., Jeffries, P., Todero, C., Rich, J., Knecht, P., et al., 2017, ‘The academic-practice gap: Strategies for an enduring problem’, *Nurse Forum* 53(1), 27–34.	Explore contemporary practices bridging the academic practise gap.	Review of literature	Simulation with appropriate guidelines and scenarios can be used to teach skills in safe environments. Learner-centred approaches to be used to engage students in their own skills development and evidence-based standards to be used to evaluate students’ competencies. Allocate students to professional nurses in clinical units for the application of theory in practice. Lecturers to collaborate with clinical staff to support clinical learning. Students to feel part of clinical teams for theory-practice application. Strong leadership in clinical and education institutions are needed to support the sharing of knowledge and human resources.	Incorporate different student-centred educational strategies to improve students’ communication skills, nursing skills and knowledge to support theory practice integration (simulation learning, flipped classroom, concept mapping and journaling). Support students to integrate learned knowledge in the clinical setting. Integrate collaborative education and practice strategies to enhance student-centred learning for support of theory practice integration and stimulation of professional growth and learning (preceptorship model, dedicated educational units within hospitals). Building a successful collaborative partnership between nursing education institutions and clinical facilitators in a hospital setting through strong leadership, clear structures and processes, an open communication system and mutual commitment to improving trust relationships.
Iseler, J., Wehrwein, T. & Jensen, C., 2019, ‘A Model of academic and service partnership focused on the clinical nurse specialist’, *Journal of Nursing Administration* 49(6), 294–296.	This article describes an innovative approach to building a partnership through the joint appointment of a full-time faculty member in a contracted clinical nurse specialist position in a community hospital.	Discussion paper	Through a partnership, lecturers with PhDs were appointed in clinical settings to improve patient care, support students and professional nurses.	The collaboration of lecturers and clinical facilitators need to be planned with clear goals and expectations, resource allocation and building of trusting relationships. Implementation of evidence-based projects to improve quality care in the hospital setting should be encouraged and supported by lecturers.
Kleinpell, R.M., Faut-Callahan, M., Carlson, E., Llewellyn, J. & Dreher, M., 2015, ‘Evolving the practitioner-teacher role to enhance practice-academic partnerships: A literature review’, *Journal of Clinical Nursing* 25(5–6), 708–718.	To review the development of the practitioner-teacher role and its use in advancing clinical nursing.	Exploratory literature review	The establishment of an organisational structure is the cornerstone for collaboration between a nursing education institution and a clinical setting. The main focus is on the integration of theory and practice. The practitioners-lecturer model supports research in practice. Quality improvement projects are implemented in hospitals to improve service quality through evidence-based practice.	Involve lecturers and clinical facilitators in research projects to address problems in the clinical setting and to implement evidence-based practice. It might lead to an improved student learning environment, quality care of patients and research culture in hospitals. Promote working relationships between lecturers and clinical facilitators. Clinical facilitators and lecturers are co-responsible for the improvement of practice and the integration of theoretical knowledge in hospital settings.
O’Neal, P.V., McClellan, L.C. & Jarosinski, J.M., 2016, ‘A new model in teaching undergraduate research: A collaborative approach and learning cooperatives’, *Nurse Education in Practice* 18, 80–84.	This study aimed to apply the Collaborative Approach and Learning Cooperatives (CALC) Model in an undergraduate nursing research course.	Descriptive pilot quality improvement project; University and 1 acute care hospital setting; 75 junior level students	Hospital managers and faculty designed a research project for undergraduate nursing students. The project promoted new working relationships between service partners, nurses and students. Nursing managers became involved in the teaching and evaluation of students. Nursing managers as part of the evaluation team may motivate students to place more effort into learning activities.	Collaboration of healthcare services and nursing education institutions should encourage staff and nursing students to solve clinical problems through research applications. Using the research process to improve practice should be encouraged as nursing education institutions, healthcare services, and students benefit. It provides opportunities for students to develop research skills and improve practice.
Sadeghnezhad, M., Nabavi, F.H., Najafi, F., Kareshki, H. & Esmaily, H., 2018, ‘Mutual benefits in academic-service partnership: An integrative review’, *Nurse Education Today* 68, 78–85.	Identify the mutual benefits of academic service partnership.	Integrative review; 28 articles	Synergy in training imply: Educational capacity development, better transition from student role to professional role, staff development, education improvement, curriculum improvement, access to shared resources, production and application of beneficial knowledge into practice, research conditions improvement, development of practical and useful interactions between nursing education institution and healthcare service.	The establishment of an effective academic-service partnership will lead to: Capacity building of academic and service staff; Curriculum improvement to support the services and student’s needs; Graduate students that have the required capabilities and skills; and sharing of financial and human resources. The development of collaborative relationships can be responsible for mutual research projects to help solve hospital service clinical problems and applying of research knowledge.
Shoghi, M., Sajadi, M., Oskuie, F., Dehnad, A. & Borimnejad, L., 2019, ‘Strategies for bridging the theory-practice gap from the perspective of nursing experts’, *Heliyon Science Direct* 5(9), 1–6.	To explore the perspective of both nursing experts in education and clinical setting about strategies for coordinating education and clinical performance in nursing to help bridge this gap.	Qualitative study; Semi-structured individual interviews and focus group interviews; Lecturers with minimum qualification with PhD degree and 5 years as an educator, nursing managers and educational supervisors	The curriculum needs to portray caring theories and models that students can use to evaluate their own competencies. What is taught in class should correspond with what happens in practice. Curriculum content needs to simulate practice and change the clinical environment. It is important to support the empowerment of lecturers and clinical staff to enable lecturers to discuss aspects of practical experience during theoretical classes and lecturers should teach clinical staff research methodologies. Clinical guidelines need to be designed to fit the local situation and support theory-practice integration.	Design a culturally conducive curriculum to support students in understanding and clarification of their values and belief systems to better integrate theory and practice. Include innovative teaching strategies for example, action research projects and flipped classrooms, and adapt theory content to what is happening in hospital services to enhance theory-practice integration. Creating an appropriate and clear clinical framework to support the use of scientific evidence. Promote sharing of knowledge and re-orientation of staff from both systems with discussion sessions and workshops, where academic and clinical speakers have equal opportunities to share their knowledge and skills. Implementing clinical guidelines in all healthcare services to standardised nursing care.
Yi, Y.J., Lee, H. & Park, K., 2020, ‘The role of academic-practice partnerships from perspectives of nursing students: A cross-sectional study’, *Nursing Education Today* 89, 1–8.	To identify the role of academic-practice partnerships from the perspective of nursing students.	Cross-sectional research study; Questionnaire was used; 242 fourth-year nursing students; Nursing college in Korea	All lecturers and clinical facilitators should share the same educational philosophy. Nursing students need to be aware of the partnership between lecturers and clinical facilitators. The students need to be encouraged to interact with the lecturers and clinical facilitators in order to develop their skills.	Create a partnership with a shared educational philosophy, clear goals and practical content to ensure teamwork. The development of lecturers and clinical facilitators is vital as they support students to develop as evidence-based practitioners. Establish standards for clinical facilitation.

In phase two of the study, a descriptive qualitative study through focus group discussions (FGDs) was done to contextualise the drafted collaboration guidelines to fit the unique circumstances of the designated nursing education institution and associated healthcare services.

## Setting

The study was conducted at one nursing education institution and its associated healthcare services in the Gauteng province in South Africa. The nursing education institution is accredited with the South African Nursing Council (SANC) for the Diploma in Nursing Science (R171 of 2013) leading to registration with the SANC as general nurses.

### Study population and sampling

The study population consisted of 10 theoretical lecturers who were responsible for theoretical teaching and 10 clinical facilitators for clinical training. Participants were purposively selected and invited to FGDs. Nine theoretical lecturers and 10 clinical facilitators participated in four focus groups discussing the contextualisation of the collaboration guidelines. Refer to Table 2 for the participants’ demographic information.

**TABLE 2:** Participants’ demographic data and qualifications status.

Participants	Nursing qualification	Experience in nursing indicated in years	Experience in nursing education indicated in years
TLP 1	Postgraduate Diploma in Nursing Education	13	9
TLP 2	B Cur and BA of Social Science	14	6
TLP 3	B Cur	10	7
TLP 4	M Cur, PhD	40	2
TLP 5	B Cur	11	5
TLP 6	D Litt et Phil	43	11
TLP 7	M Cur	34	7
TLP 8	M Cur	33	15
TLP 9	B Cur	10	1
CFP 1	B Cur	45	15
CFP 2	Diploma in Nursing Education, Management	25	17
CFP 3	B Cur Iet A; OHS	13	1
CFP 4	B Cur Honours PHC	20	6
CFP 5	B Cur	35	6
CFP 6	B Cur	17	4
CFP 7	B Cur	15	8
CFP 8	PhD	28	15
CFP 9	B Cur Iet A, Adv ICU diploma	13	1
CFP 10	B Cur	21	6

*Source:* Bester, E., Van Wyk, N.C. & Maree, C.M., 2023, ‘Collaboration guidelines for a designated nursing education institution and associated healthcare services’, Masters dissertation, University of Pretoria repository.

TLP, Theoretical lecturer participant; CFP, Clinical facilitator participant, BCur, Baccalareus Curationis; BCur I et A, Baccalareus Curationis (Instructionis et Administrare); MCur, Magister Curationis; PHC, Primary Health Care; Adv ICN, Advanced Intensive Care Nursing; OHS, Occupational Health Science; D. Litt et Phil, Doctor of Literature and Philosophy.

### Data collection

Draft guidelines were contextualised through four FGDs at a suitable venue at the nursing education institution. The discussions were audio-recorded with the participants’ permission. The first author facilitated the discussions, and participants provided input by discussing each guideline, deleting irrelevant ones, and adapting others to their specific collaboration needs. Four FGDs were necessary before the participants agreed upon the set of contextualised guidelines. The first author made field notes of the participants’ interaction that was not audio-recorded.

### Data analysis

The audio-records of the FGDs were transcribed and together with the field notes were read several times by the first author to familiarise herself with the content. Themes were identified and coded, grouped into sub-categories and categories. The categories and sub-categories informed the contextualisation of the collaboration guidelines between the nursing education institution and healthcare services.

### Trustworthiness of the findings

The researcher involved theoretical lecturer and clinical facilitator participants in contextualising the draft guidelines. Their expertise and experiences were valuable to adjust the guidelines to suit the unique circumstances of the designated nursing education institution and associated healthcare services.

### Ethical considerations

A study information leaflet explaining the aims and research process, with draft guidelines, was provided to the participants who voluntarily gave informed consent before participating in the FGDs. They were informed they are allowed to withdraw from the research at any time without negative consequences. Confidentiality was emphasised during the discussions. The study proposal was approved by the Faculty of Health Sciences Research Ethics Committee at the University of Pretoria (Ref 670 of 2020). Permission was obtained from the management of the healthcare services and nursing education institution where the research was conducted.

## Results

The participants through their input during FGDs contextualised the collaboration guidelines. Their discussion to contextualise the draft guidelines is reflected in categories and sub-categories (see Table 3).

**TABLE 3:** Categories and sub-categories.

Categories	Subcategories
Maintain a healthcare service and nursing education institution partnership	Managerial support of the partnership An integrated unit for clinical teaching Fusion of roles in theoretical and clinical teaching
Maintain bilateral communication	Maintain an open communication structure between clinical facilitators and theoretical lecturers
Maintain cooperation between theoretical lecturers and clinical facilitators in evidence-based practice	Evidence-based projects for clinical problems Knowledge sharing for theory-practice integration
Intensify innovation in teaching, learning and assessment processes	Cooperating with the implementation and execution of teaching and learning within an operational plan Sharing of innovative teaching strategies Participation in assessment design and processes
Build an environment conducive to theory-practice integration	Support of newly appointed theoretical lecturers and clinical facilitators through mentorship Empowerment of theoretical lecturers and clinical facilitators

### Maintain a healthcare service and nursing education institution partnership

Shared values and goals enhance collaboration for improved teamwork:

‘Teamwork should include academic staff and hospital management staff. They should know what values and ethics we strive for so that there is better integration between hospital management staff and us.’ (TLP4, M Cur, PhD, 40 years)

Relationships between facilitators and lecturers foster shared responsibility, commitment and connectivity in the partnership, vital for team development and expanding teamwork:

‘Relationship is the most important thing we are having.’ (CFP4, B Cur Honours PHC, 20 years)

Clinical facilitators require knowledge of the theory content taught to support and align theory-practice integration in hospitals, bridging the gap between theory taught in class rooms, and its application in the hospital environment:

‘Clinical facilitators should know the textbook content; should know the theoretical questions to facilitate better at hospital.’ (TLP6, D Litt et Phil, 43 years)

‘Clinical facilitators should know the theoretical content to improve theory-practice integration.’ (CFP6, B Cur, 17 years)

### Maintain bilateral communication

Implementing an organised, goal-driven communication plan is crucial for facilitating regular sharing of relevant information among clinical facilitators and theoretical lecturers. This ensures effective collaboration and supports the integration of theory and practice:

‘Clarity of expectations of communication is required from both clinical and theory.’ (CFP2, Diploma in Nursing Education, Management, 25 years)

### Maintain cooperation between theoretical lecturers and clinical facilitators in evidence-based practice

Collaborative leadership and teamwork between clinical facilitators and theoretical lecturers are essential for implementing evidence-based practice, enhancing academic staff skills, and facilitating the transfer of knowledge between hospitals and nursing education institutions. This joint effort addresses the theory-practice gap, promotes the development of evidence-based practice, and fosters a culture conducive to learning:

‘Collaboration and shared leadership that clinical facilitators and lecturers should identify the need in the clinical field for an assignment and clinical project in the research, to fill the gap.’ (TLP2, B Cur and BA of Social Science, 14 years)

Sharing evidence-based practice processes and knowledge generated in hospitals may motivate staff and students to improve their clinical practice and advance their knowledge. Utilising evidence-based practice may enhance the students’ learning environment and improve theory-practice integration:

‘Manage a positive working environment by strengthening the lecturers and clinical facilitator’s skills in teaching and learning through sharing of evidence-based practices.’ (TLP8, M CUR, 33 years)

### Intensify innovation in teaching, learning and assessment processes

The implementation of an operational plan by the nursing education institution management may be instrumental in supporting the integration and alignment of theory and practice. Such a plan may effectively structure the collaboration between clinical facilitators and theoretical lecturers:

‘Operational plan has a structured clinical for learning programme for the day.’ (CFP6, B Cur, 17 years)

‘Academic staff is responsible for planning, implementation and execution of operational programme.’ (TLP 7, M CUR, 34 years)

Innovative and creative teaching and learning methods that prioritise student-centred methods are crucial for accommodating diverse types of nursing students in both classroom and hospital settings. Clinical facilitators and theoretical lecturers should therefore use innovative methods to foster critical thinking, clinical judgement, and reflective thinking, effectively addressing the unique learning needs of the students:

‘We need to find ways to improve our teaching, like, seminars, journal clubs on teaching to develop us or enhance our skill in teaching.’ (TLP5, B Cur, 11 years)

‘We need to be equipped for the types of students with different learning styles, we need to be able to accommodate them in the teaching and learning environment.’ (CFP10, B Cur, 21 years)

Active participation is a core value for team success and achieving desired outcomes. To ensure competent nursing students, clinical facilitators must actively engage in the design of clinical assessments:

‘We need to be involved or participate in designing and evaluating. It’s about participation.’ (TLP4, M Cur, PhD, 40 years)

### Build an environment conducive to theory-practice integration

Mentorship programmes play a vital role in fostering a sense of belonging within a group, which in turn supports theory-practice integration. It is crucial for clinical facilitators and theoretical lecturers, to possess mentorship skills. Therefore, providing mentorship training is essential to ensure adequate support for new staff and foster the development of relationships that enhance collaboration:

‘Mentoring is a skill, need to be knowledgeable on what mentoring is about. Mentoring is a connection, relationship and time.’ (TLP4, M Cur, PhD, 40 years)

The participants agreed that theoretical lecturers and clinical facilitators need to be experts in theory-practice integration, to support students with theory-practice integration:

‘Clinical facilitators and lecturers need to become specialists in a discipline for teaching and skill.’ (TLP4, M Cur, PhD, 40 years)

## Discussion

Effective collaboration between nursing education institutions and healthcare services’ management is crucial. Each partner should have defined responsibilities, with coordination overseen by designated persons. A structure is needed to facilitate the creation, translation and application of knowledge, benefiting student learning and patient care (Gilliss et al. 2021:4,5). The appointment of clinical coordinators can enhance cooperation between theoretical lecturers and clinical facilitators, supporting students’ skills development (Munangatire & McInerney 2022:2).

A shift is necessary for theoretical lecturers and clinical facilitators to transition from individual ownership of students’ teaching to a partnership model of student support (Huston et al. 2018:32). Partnerships foster commitment, clarify roles and responsibilities, and facilitate the integration of theory and practice in the hospital setting. By improving the understanding of roles and responsibilities, coordination between clinical facilitators and theoretical lecturers can be enhanced, leading to better team outcomes and theory-practice integration (Munangatire & MCinerney 2022:2). Clinical facilitators play a crucial role in theory-practice integration when they apply theoretical knowledge during clinical training of students. Sharing theoretical class content with clinical facilitators promotes cooperative teaching in the hospital, facilitating theory-practice integration (Ryan & McAllister 2020:2). Open, trustful and supportive communication is imperative for improving cooperation (Bvumbwe 2016:320). When clinical facilitators and theoretical lecturers engage in collaborative thinking and reasoning, their collective output enhances students’ learning and skills development (Askari et al. 2020:2).

Evidence-based practices play a vital role in addressing patient care challenges, generating new knowledge, and improving the learning environment for students (Ramis et al. 2019:11). The implementation of evidence-based practice also encourages the professional growth of theoretical lecturers and clinical facilitators by enhancing their clinical skills. Integrating evidence-based practice into routine patient care should be a key focus of the partnership between nursing education institutions and healthcare services. This integration aims to enhance patient outcomes and improve the quality of care provided (Kleinpell et al. 2016:706).

An operational plan is crucial in facilitating the teaching and learning process both at the nursing education institution and in hospitals, aiming to align theory teaching with practical training. Such plans promote the acquisition of clinical skills while integrating theory, minimising the theory-practice gap and fostering students’ professional development (Ngozika Ugwu et al. 2023:11). Strategic clinical planning activities are essential for supporting the operational plan and ensuring effective theory-practice integration for students (Hoda Ahmari et al. 2021:265).

Innovative assessment strategies should be created by theoretical lecturers and clinical facilitators (Van Wyngaarden, Leech & Coetzee 2019:4). Joint assessment practices between them can standardise expectations and promote understanding. Collaborative decision-making in assessment can prevent inconsistencies (Dev et al. 2020:1135).

Mentorship is essential for enhancing teaching practices, skills and collaboration within the academic team (Glover et al. 2021:272). It provides support for clinical facilitators and theoretical lecturers as they transition into their roles as educators, fostering excellence (Huston et al. 2018:32). Theoretical lecturers and clinical facilitators should focus on improving their teaching and learning skills to enhance the learning environment. Sharing innovative student-centred strategies and skills such as student feedback, mentoring, engaging in research activities and embracing management diversity is essential for fostering teamwork and positive relationships (Mallek & El-Hosany 2020:379). These actions contribute to an enhanced teaching and learning experience.

### Context-specific guidelines to improve collaboration and narrow the theory-practice gap in nursing education

**FIGURE 2:**
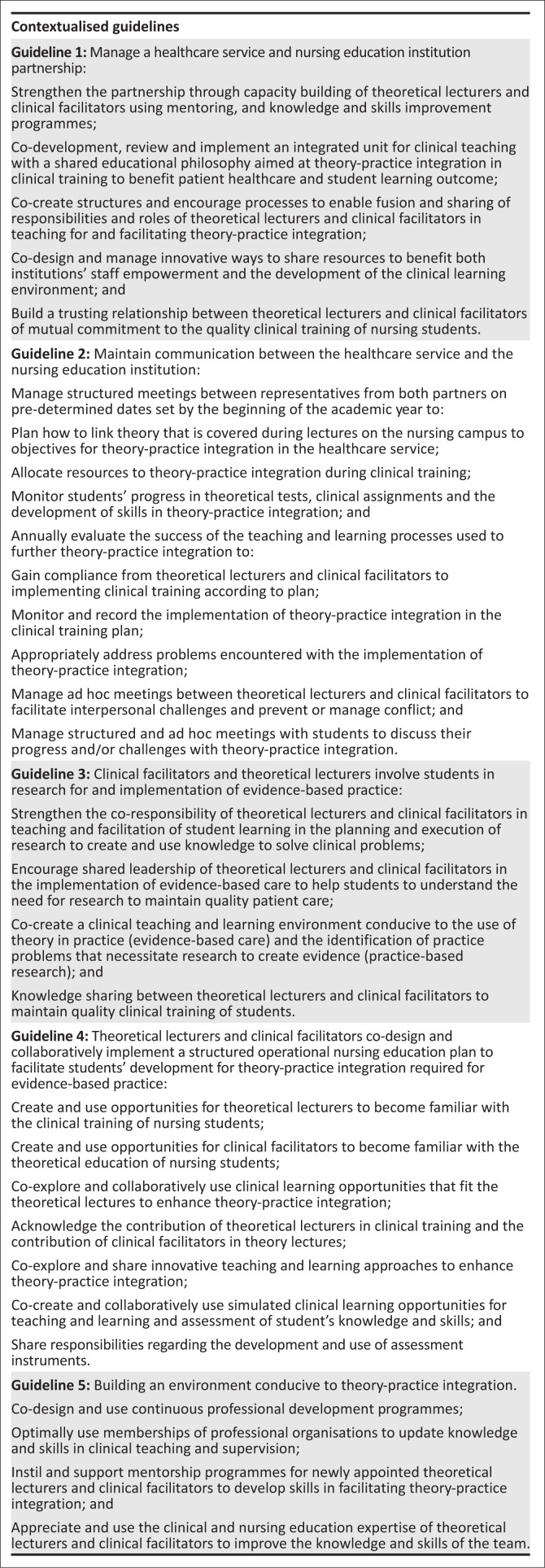
Contextualised guidelines.

The guidelines are the outcome of a thorough literature review to obtain published research evidence, the identification of draft guidelines from the evidence, and a contextualisation with the input of theoretical lecturers and clinical facilitators who may benefit from its implementation. The categories and subcategories as the product of the analysis of the data of the FGDs feature in the final set of collaboration guidelines. Refer to Figure 2 for description of the contextualised guidelines.

### Strengths and limitations

Although the study was conducted at one nursing education institution and its associated healthcare services only, the authors described the process of the development and contextualisation of the guidelines thoroughly to enable researchers to repeat the research to benefit other nursing education institutions and associated healthcare services.

### Implications for nursing education institutions and healthcare services

The guidelines can be adjusted to suit the specific context of the nursing education institution and the associated healthcare services, and can be used to improve collaboration between theoretical lecturers and clinical facilitators to benefit students’ endeavours to integrate theory and practice. Effective collaboration and relationships between clinical facilitators and theoretical lecturers can narrow the gap that often exists between nursing education institutions and healthcare services to advance optimal support of theory-practice integration.

## Conclusion

The challenges faced in applying nursing theory to clinical practice necessitate evidence-based interventions derived from the research literature. In this study, the researcher, with the guidance of supervisors, conducted a literature review to gather evidence. Based on this review, guidelines were formulated and contextualised through FGDs involving clinical facilitators and theoretical lecturers. The implementation of these guidelines has the potential to support nursing students in effectively applying nursing theory in practice.
